# Cardiac rehabilitation influences serum myokine levels in patients after acute coronary syndrome: the randomised CARDIO-REH study

**DOI:** 10.1038/s41598-025-22897-0

**Published:** 2025-11-06

**Authors:** Damian Skrypnik, Katarzyna Skrypnik, José Casaña Granell, Dawid Woszczyk, Joanna Suliburska

**Affiliations:** 1 Department of Family Medicine, Poznań University of Medical Sciences, Przybyszewskiego St. 49, Poznan, 60-355 Poland; 2 Department of Human Nutrition and Dietetics, Poznan University of Life Sciences, Wojska Polskiego St. 31, Poznan, 60-624 Poland; 3 Department of Physiotherapy, Exercise Intervention for Health Research Group (EXINH-RG), University of Valencia, C/Gascó Oliag 5, Valencia, 46010 Spain; 4 Student Scientific Association of Lifestyle Medicine, The Student Scientific Society of Poznan University of Medical Sciences, Poznan University of Medical Sciences, Poznan, Poland; 5 University Clinical Hospital in Poznan, Przybyszewskiego St. 49, Poznan, 60- 355 Poland

**Keywords:** Cardiac rehabilitation, Myokines, Myostatin, Follistatin, Apelin, Follistatin-related protein 1, Acute coronary syndrome, Physiology, Cardiology, Medical research, Risk factors

## Abstract

The role of myokines as a link between cardiac rehabilitation (CR) and cardiovascular benefits in patients recovering from acute coronary syndrome (ACS) is important but not well understood. We investigated the effect of CR on circulating levels of myostatin, follistatin, apelin, and follistatin-related protein 1 (FSTL1) in post-ACS patients. A total of 110 patients underwent a 2-week CR programme (group S) and were compared with 110 non-CR patients (group K). In group S, blood pressure, heart rate, anthropometrics, body composition, and serum myokine levels were measured at baseline and post-CR; in group K, these were assessed once. After CR, apelin, myostatin, and FSTL1 increased in group S. Apelin and myostatin were higher in group S post-CR compared with group K, while follistatin remained higher in group K at both time points. FSTL1 was initially higher in group K but increased post-CR in STEMI patients only; myostatin increased in NSTEMI patients. Apelin increased in STEMI patients. Apelin and myostatin levels were independent of cardiovascular risk factors. Post-CR, follistatin correlated inversely with diastolic pressure; FSTL1 was related to fat tissue, muscle mass and body mass index. CR modulates key myokines and differential myokine responses in STEMI vs. NSTEMI patients support the need for personalised rehabilitation strategies. ClinicalTrials.gov registration number: NCT03935438.

**Supplementary Information:**

The online version contains supplementary material available at 10.1038/s41598-025-22897-0.

## Introduction

In 2021, ischaemic heart disease (IHD) led to as many as 9,960,000 deaths. This translated into 196 × 10^6^ disability-adjusted life years (DALYs), which means that the milestone of 200 × 10^6^ DALYs is fast approaching. It is undeniable that IHD remains the top cause of cardiovascular deaths^1^. Currently, 1.72% of the global population suffers from IHD, meaning that approximately 126 million people affected. Predictions show that by 2030 this rate will rise to 1.845%^2^. The annual cost of therapy after acute coronary syndrome (ACS), depending on the method of treatment, ranges from $34,087 to $86,914^3^.

Low physical activity was responsible for 684,000 deaths and over 11 × 10^6^ DALYs attributed to cardiovascular diseases in 2021^1^. This strongly justifies the crucial role of cardiac rehabilitation in the prevention and therapy of cardiovascular diseases^4–7^. Physical activity during cardiac rehabilitation in patients recovering from ACS is an essential intervention; it results in a more favourable health prognosis compared with patients who do not participate in cardiac rehabilitation after ACS^8,9^. Cardiac rehabilitation diminishes the frequency of major adverse cardiac events (MACE; relative risk [RR] 0.49), the risk of ACS recurrence (RR 0.63) and the cardiac death rate (RR 0.40). As a result, the mortality hazard ratio is 0.47 in patients after cardiac rehabilitation compared with patients who do not receive such intervention^10^. Thus, cardiac rehabilitation is recommended in individuals after ACS in the line with the result of their cardiac stress test (CPX)^9,11^, which was approved by the European Society of Cardiology (ESC) as a high-quality and broadly validated instrument to determine the cardiovascular risk in patients with IHD^12^.

Myokines are cytokines and other peptides synthesised and secreted by muscle tissue and able to exert endocrine, paracrine and autocrine effects^13^. There has been a rapid increase in scientific interest regarding the role of myokines in human biology and medicine. Myokines enable communication between muscles and other tissues and organs, including the cardiovascular system, liver, gut, skin and adipose tissue, and regulate an extensive range of metabolic processes, endothelial cell function, glucose and lipid metabolism and hypertrophy^13^. The elucidation of how myokines operate may lead to a better understanding of the role of muscular system in the treatment strategies of cardiovascular diseases, including IHD, and to the development of new therapeutic approaches and improvement of those already used^13^. The most important myokines related to cardiovascular system function are myostatin^14^, follistatin^15,16^, apelin^17^ and follistatin-related protein 1 (FSTL1)^18,19^.

Apelin is a pleiotropic peptide^20^ involved in angiogenesis, fluid homeostasis, energy metabolism and cardiovascular system functioning. Increased apelin expression lowers blood pressure, elevates cardiac output and reduces hypertrophy and oxidative stress. Apelin plays important role in pathologies such as hypoxia-related diseases and heart failure, in which it shows a cardioprotective effect by defending the myocardium against infarction. In hypoxic conditions, apelin counteracts apoptosis and enhances the activity of antioxidant enzymes^17^. Data on the effect of exercise on blood apelin levels are inconsistent: while in sportsmen a bout of maximum exercise increases the blood apelin level^21^, in patients with obesity, aerobic and resistance training decreases it^22,23^.

Myostatin was the first factor defined as myokine. It belongs to the transforming growth factor β (TGF-β) superfamily and is a negative regulator of myogenesis via an autocrine function^24^. Significant myostatin synthesis is observed in the skeletal muscles as well as the heart. Myostatin is upregulated under some pathological cardiac conditions, including myocardial infarction and heart failure^14,22,25^. In the heart, myostatin stabilises the metabolic status and energy homeostasis of cardiomyocytes and prevents cardiac hypertrophy^14^. Furthermore, myostatin secreted in the heart has been identified as endocrine mediator of heart-induced muscle wasting and atrophy, which in case of cardiac stress induced by cardiovascular disease supports elevated blood circulation. However, if long lasting, then this myostatin-mediated cardiac damage appears to be maladaptive^26^. The blood myostatin level is reduced in response to training^27,28^.

Follistatin is a glycoprotein^29^ that acts as an antagonist of myostatin^30^. Recently, the cardioprotective properties of follistatin have been revealed. In the heart, follistatin counteracts cardiac fibrosis and reduces the production of reactive oxygen species (ROS). It can also decrease the level of TGF-β superfamily members and promote hypertrophy of the heart, but without changes in atrial natriuretic peptide (ANP) expression^15^. Follistatin is downregulated in the left ventricle of the heart after myocardial infarction, a reduction that is associated with myocardial fibrosis and heart failure following myocardial infarction. Interestingly, follistatin protein and messenger ribonucleic acid (mRNA) levels after myocardial infarction are also decreased in skeletal muscles^16^. The blood follistatin level is increased during exercise^31,32^.

FSTL1 is a glycoprotein produced in cardiomyocytes and skeletal muscles^33^ that enhances the proliferation of cardiac muscle cells and decreases apoptosis, thus preventing cardiac rupture in IHD conditions^18^. Moreover, it participates in cross-talk between cardiomyocytes and cardiac fibroblasts in ischaemic conditions^18^. FSTL1 shows cardioprotective and antiapoptotic properties^19^ and deters vascular hypertrophy^34^. Its blood concentration is elevated in patients after ACS and in patients with chronic heart failure^33^. In animal models of myocardial infarction, aerobic training increases the blood FSTL1 level and its expression in skeletal muscles and the myocardium^34^.

The influence of cardiac rehabilitation on blood myostatin, follistatin, apelin and FSTL1 levels and the link between possible alterations in these myokines and cardiovascular system function in humans after ACS remains unknown. Physical training implemented during cardiac rehabilitation in patients recovering from ACS involves massive, regular engagement of the muscular system for a certain period of time. This phenomenon leads to two hypotheses: first, this physical effort influences the secretion of these four myokines into the blood. Second, alterations in the blood levels of these four myokines represents a significant ‘puzzle piece’ regarding cross-talk between muscles and the cardiovascular system during cardiac rehabilitation, with amelioration of cardiovascular system function. Although it has been almost 30 years since the first myokine was identified, these two hypotheses have not yet been tested appropriately. In the light of rapidly growing IHD epidemic, there is great scientific interest in this area. In the present study, we aimed to investigate the influence of a 2-week cardiac rehabilitation programme on circulating myostatin, follistatin, apelin and FSTL1 levels in patients after ACS. We also explored the subtle cross-talk between serum myokine level changes and selected classical parameters of cardiovascular risk. This study is novel due to its innovative scientific approach, searching to understand the basis of beneficial effect of cardiac rehabilitation on the cardiovascular system in a population of patients who are recovering from ACS.

## Materials and methods

### Study design

The trial was designed as a randomised, interventional, prospective and comparative study. The study protocol was approved by the Bioethics Committee, Poznan University of Medical Sciences (no. 476/19 with amendments). The study was performed in accordance with relevant guidelines and regulations, including The Declaration of Helsinki (1975 revision with amendments) and regulations of Poznan University of Medical Sciences. Written informed consent was obtained from all subjects and/or their legal guardian(s). The study was conducted in Poznan University of Medical Sciences, Poznan, Poland. The study lasted from April 2019 to September 2023. The CONSORT recommendations were implemented. The trial has been registered at ClinicalTrials.gov under NCT03935438 (the first registration date 02/05/2019; https://clinicaltrials.gov/study/NCT03935438?term=NCT03935438&rank=1).

### Statistical analysis

Statistical analyses were performed using Statistica version 13 (StatSoft, Tulsa, OK, USA). Data distribution was assessed with the Shapiro–Wilk test. Continuous variables are presented as median [Q1- first quartile; Q3- third quartile]. Baseline characteristics of the study and control groups (Table 1) are reported descriptively; these comparisons were not adjusted for multiple testing, as they were intended to describe the sample rather than to test study hypotheses. For the main analyses (Tables 2, 3, 4 and 5), within-group changes before and after cardiac rehabilitation were compared using paired Student’s t-tests or Wilcoxon signed-rank tests, depending on normality. Between-group comparisons were performed with independent Student’s t-tests or Mann–Whitney U tests. Fisher’s exact test was used for categorical variables. To control for multiple testing in the main outcomes, p-values were adjusted using the Bonferroni procedure.

**Table 1:** Baseline characteristics of group S and K.

Parameter	Group S	Group K	*p*-value
n	99	100	
Age [years]	65 [59; 71]	66 [58; 74]	0.6730 *
Height [cm]	168.0 [163.0; 177.0]	168.0 [161; 174]	0.4405 *
Body mass [kg]	76.5 [67.3; 86.9]	83.7 [72.3; 99.0]	**0.0073 #**
BMI [kg/m2]	26.6 [23.9; 30.4]	29.4 [25.7; 33.2]	**0.0018 #**
Apelin [ng/ml]	576.14 [383.48; 823.92]	646.83 [373.40; 902.26]	0.5481 #
SBP [mmHg]	126.0 [112.0; 142.0]	133.0 [119.0; 150.0]	**0.0360** *
DBP [mmHg]	74.0 [68.0; 82.0]	74.0 [67.0; 86.0]	0.5277 #
HR [bpm]	68.0 [62.0; 75.0]	67.0 [63.0; 74.0]	0.8857 #

Data are presented as median [Q1; Q3]. *BMI* Body mass index, *bpm* Beats per minute, *DBP* Diastolic blood pressure, *HR* Heart rate, *Q1* First quartile, *Q3* Third quartile, *SBP *Systolic blood pressure, #Mann-Whitney test, *t-student test for independent variables. Significant p-value is bolded.

**Table 2:** Anthropometric, body composition analysis, resting blood pressure and resting heart rate results.

Parameter	Group	Median [Q1; Q3]	*p*-value	
Body height [cm]	S (I)	168.0 [163.0; 177.00]	S (I) vs. K	0.4405 *
	K	168.0 [161.0; 174.0]		
Body mass [kg]	S (I)	76.5 [67.3; 86.9]	S (I) vs. S (II)	**0.0007** ##
	S (II)	77.5 [66.8; 87.5]	S (I) vs. K	**0.0073** #
	K	83.7 [72.3; 99.0]	S (II) vs. K	0.0231 #
BMI [kg/m2]	S (I)	26.6 [23.9; 30.4]	S (I) vs. S (II)	**0.0006** ##
	S (II)	27.3 [23.7; 31.2]	S (I) vs. K	**0.0018** #
	K	29.4 [25.7; 33.2]	S (II) vs. K	**0.0102** #
HC [cm]	S (I)	102.0 [97.0; 109.0]	S (I) vs. S (II)	0.5811 ##
	S (II)	101.0 [98.0; 107.0]	S (I) vs. K	0.0237 #
	K	106.0 [99.0; 115.0]	S (II) vs. K	0.0368 #
WC [cm]	S (I)	98.0 [93.0; 108.0]	S (I) vs. S (II)	0.7389 ##
	S (II)	99.0 [95.0; 107.0]	S (I) vs. K	**0.0002** #
	K	106.0 [98.0; 116.0]	S (II) vs. K	**0.0027** *
%FTC [%]	S (I)	32.1 [24.4; 37.6]	S (I) vs. S (II)	0.0237 **
	S (II)	32.7 [25.8; 40.3]	S (I) vs. K	0.0194 *
	K	35.4 [28.6; 43.5]	S (II) vs. K	0.1169 *
FTC [kg]	S (I)	22.55 [18.70; 30.30]	S (I) vs. S (II)	0.5059 ##
	S (II)	25.70 [20.20; 31.25]	S (I) vs. K	0.0229 #
	K	30.50 [20.40; 41.10]	S (II) vs. K	0.0876 #
FFM [kg]	S (I)	52.60 [41.80; 61.50]	S (I) vs. S (II)	**0.0029** **
	S (II)	52.90 [42.70; 59.90]	S (I) vs. K	0.6658 *
	K	51.10 [44.20; 60.90]	S (II) vs. K	0.8979 *
MM [kg]	S (I)	29.30 [23.70; 34.80]	S (I) vs. S (II)	**0.0006** ##
	S (II)	29.20 [23.00; 32.90]	S (I) vs. K	0.7961 #
	K	28.40 [23.70; 34.40]	S (II) vs. K	0.7053 #
BMR [kcal]	S (I)	1525 [1326; 1711]	S (I) vs. S (II)	**0.0035** **
	S (II)	1512 [1294; 1662]	S (I) vs. K	0.7998 *
	K	1475 [1325; 1702]	S (II) vs. K	0.8969 *
SBP [mmHg]	S (I)	126.0 [112.0; 142.0]	S (I) vs. S (II)	**< 0.0001** **
	S (II)	120.0 [109.0; 133.5]	S (I) vs. K	0.0360 *
	K	133.0 [119.0; 150.0]	S (II) vs. K	**< 0.0001** *
DBP [mmHg]	S (I)	74.0 [68.0; 82.0]	S (I) vs. S (II)	**0.0121** ##
	S (II)	72.0 [65.5; 78.0]	S (I) vs. K	0.5277 #
	K	74.0 [67.0; 86.0]	S (II) vs. K	**0.0153 #**
HR [bpm]	S (I)	68.0 [62.0; 75.0]	S (I) vs. S (II)	0.9354 ##
	S (II)	67.5 [60.0; 74.5]	S (I) vs. K	0.8857 #
	K	67.0 [63.0; 74.0]	S (II) vs. K	0.5639 #

Data are presented as median [Q1; Q3]. *%FTC* Percentage fat tissue content, *BMI* Body mass index, *BMR* Basal metabolic rate, *bpm* Beats per minute, *DBP* Resting diastolic blood pressure, *FFM* Fat-free mass, *FTC* Mass fat tissue content, *HC* Hip circumference, *HR* Resting heart rate, *MM *Muscle mass, *Q1* First quartile, *Q3* Third quartile, *SBP* Resting systolic blood pressure, *WC* Waist circumference.

(I) value before intervention, (II) value after the intervention.

#Mann-Whitney test, *t-student test for independent variables, ## Wilcoxon test, **t-student test for dependent variables, α = 0.0167 – Bonferroni correction. Significant p-value is bolded.

**Table 3:** Serum concentration of biochemical parameters.

Parameter	Group	Median [Q1; Q3]	*p*-value	
Apelin [ng/ml]	S (I)	576.14 [383.48; 823.92]	S (I) vs. S (II)	**0.0007** ##
	S (II)	920.11 [417.90; 2064.20]	S (I) vs. K	0.5481 #
	K	646.83 [373.40; 902.26]	S (II) vs. K	**0.0003** #
Myostatin [ng/ml]	S (I)	1.22 [0.71; 2.76]	S (I) vs. S (II)	**0.0059** ##
	S (II)	1.37 [1.04; 8.27]	S (I) vs. K	0.6543 #
	K	1.16 [0.87; 3.13]	S (II) vs. K	**0.0129 #**
Follistatin [ng/ml]	S (I)	4.91 [3.54; 6.69]	S (I) vs. S (II)	0.2737 ##
	S (II)	4.55 [3.10; 7.07]	S (I) vs. K	**0.0002** #
	K	6.31 [4.47; 8.82]	S (II) vs. K	**0.0002** #
FSTL1 [ng/ml]	S (I)	2.13 [1.19; 3.80]	S (I) vs. S (II)	**0.0018** ##
	S (II)	3.71 [2.01; 5.28]	S (I) vs. K	**0.0002** #
	K	4.09 [2.15; 7.70]	S (II) vs. K	0.2468 #

Data are presented as median [Q1; Q3]. *FSTL1* Follistatin-related protein 1, *Q1* First quartile, *Q3* Third quartile.

(I): value before intervention; (II): value after the intervention.

# Mann-Whitney test; ##Wilcoxon test; α = 0.0167– Bonferroni correction. Significant p-value is bolded.

**Table 4:** Comparison of anthropometric, body composition analysis, resting blood pressure and resting heart rate results between STEMI and NSTEMI patients in subjects from group S.

Parameter	STEMI (*n* = 58)/ NSTEMI (*n* = 35)	Before intervention	After intervention	*p*-value
		Median [Q1; Q3]	Median [Q1; Q3]	
Body mass [kg]	STEMI	78.25 [67.50; 86.10]	77.65 [68.90; 87.50]	0.5322 ##
	NSTEMI	75.00 [67.20; 95.00]	75.70 [66.60; 84.50]	0.0442 ##
	p-value	0.9557 #	0.5646 #	
BMI [kg/m2]	STEMI	26.54 [23.87; 30.39]	26.97 [23.70; 30.75]	0.0219 ##
	NSTEMI	27.40 [23.90; 32.87]	27.50 [24.24; 32.40]	**0.0076 ##**
	p-value	0.4754 #	0.7706 #	
HC [cm]	STEMI	103.0 [98.0; 109.0]	102.0 [98.0; 109.0]	0.3263 ##
	NSTEMI	99.5 [96.5; 110.5]	100.0 [98.0; 105.0]	0.6832 ##
	p-value	0.8294 #	0.5740 #	
WC [cm]	STEMI	99.0 [92.0; 108.0]	100.0 [95.0; 107.0]	0.6795 **
	NSTEMI	97.5 [93.0; 109.5]	98.0 [94.0; 108.0]	0.3634 ##
	p-value	0.8535 #	0.6014 #	
%FTC [%]	STEMI	31.8 [26.0; 36.5]	31.7 [25.8; 37.7]	0.3036 **
	NSTEMI	33.4 [22.5; 40.5]	34.2 [26.5; 42.3]	0.0396 **
	p-value	0.8764 #	0.4184 #	
FTC [kg]	STEMI	24.00 [20.90; 29.20]	25.10 [20.35; 30.35]	0.8796 ##
	NSTEMI	22.50 [18.00; 32.30]	25.70 [20.15; 31.50]	0.2114 ##
	p-value	0.6341 #	0.9651 #	
FFM [kg]	STEMI	52.35 [45.10; 62.10]	52.90 [43.50; 62.25]	0.0690 **
	NSTEMI	51.00 [41.30; 61.50]	50.70 [41.00; 56.15]	0.0197 **
	p-value	0.3573 #	0.1969 #	
MM [kg]	STEMI	29.15 [24.50; 35.00] (mean ± SD: 29.78 ± 6.22)	29.25 [23.50; 34.45] (mean ± SD: 29.72 ± 6.43)	0.0262 **
	NSTEMI	28.95 [22.10; 34.40]	27.65 [21.85; 30.85]	**0.0035 ##**
	p-value	0.3364 #	0.1716 #	
BMR [kcal]	STEMI	1546 [1357; 1745]	1519 [1324; 1738]	0.0656 **
	NSTEMI	1418 [1244; 1640]	1409 [1245; 1579]	**0.0052 ##**
	p-value	0.1069 #	0.1009 #	
SBP [mmHg]	STEMI	122.0 [109.0; 131.0]	118.0 [110.0; 130.0]	0.0336 ##
	NSTEMI	135.0 [117.00; 148.0]	120.0 [105.0; 138.0]	0.0006 **
	p-value	**0.0026 #**	0.5196 #	
DBP [mmHg]	STEMI	74.5 [66.0; 82.0]	72.0 [67.0; 78.0]	0.1342 ##
	NSTEMI	75.00 [69.00; 83.00]	69.00 [64.00; 75.00]	0.0109 **
	p-value	0.8303 #	0.1354 #	
HR [bpm]	STEMI	67.0 [63.0; 72.0]	67.0 [60.0; 75.0]	0.6251 **
	NSTEMI	68.0 [61.0; 77.0]	68.0 [59.0; 72.0]	0.7456 ##
	p-value	0.7239 #	0.5938 #	

Data are presented as median [Q1; Q3]. *%FTC* Percentage fat tissue content, *BMI* Body mass index, *BMR* Basal metabolic rate, *bpm* Beats per minute, *DBP* Resting diastolic blood pressure, *FFM* Fat-free mass, *FTC* Mass fat tissue content, *HC* Hip circumference, *HR* Resting heart rate, *MM* Muscle mass, *NSTEMI* Non-ST- segment elevation myocardial infarction, *Q1* First quartile, *Q3* Third quartile, *SBP* Resting systolic blood pressure, *SD* Standard deviation, *STEMI *ST-segment elevation myocardial infarction, *WC* Waist circumference. # Mann-Whitney test; * t-student test for independent variables; ## Wilcoxon test; ** t-student test for dependent variables; α = 0.0125 – Bonferroni correction. Significant p-value is bolded.

**Table 5:** Comparison of serum concentration of biochemical parameters between STEMI and NSTEMI patients in subjects from group S.

Parameter	STEMI (*n* = 58)/ NSTEMI (*n* = 35)	Before intervention	After intervention	p-value
		Median [Q1; Q3]	Median [Q1; Q3]	
Apelin [ng/ml]	STEMI	509.86 [355.97; 820.33]	939.23 [569.36; 2064.20]	**< 0.0001 ##**
	NSTEMI	681.36 [388.66; 853.54]	830.66 [381.55; 2626.70]	0.0198 ##
	p-value	0.2566 #	0.4252 #	
Myostatin [ng/ml]	STEMI	1.50 [0.78; 6.36]	1.45 [1.05; 8.27]	0.4282 ##
	NSTEMI	0.98 [0.69; 2.11]	1.48 [1.04; 15.49]	**0.0004 ##**
	p-value	0.0746 #	0.6706 #	
Follistatin [ng/ml]	STEMI	5.03 [3.54; 6.05]	4.50 [3.03; 5.56]	0.0522 ##
	NSTEMI	4.45 [3.53; 7.01]	4.87 [3.03; 7.42]	0.8664 ##
	p-value	0.8896 #	0.4906 #	
FSTL1 [ng/ml]	STEMI	1.71 [1.14; 3.35]	3.58 [2.01; 6.74]	**0.0015 ##**
	NSTEMI	2.26 [1.23; 4.45]	3.75 [1.35; 4.77]	0.0716 ##
	p-value	0.2653 #	0.7432 #	

Data are presented as median [Q1; Q3].* FSTL1* Follistatin-related protein 1, *NSTEMI *Non-ST- segment elevation myocardial infarction, *Q1* First quartile, *Q3* Third quartile, *STEMI* ST-segment elevation myocardial infarction.

#Mann-Whitney test; *t-student test for independent variables; ##Wilcoxon test; **t-student test for dependent variables, α = 0.0125 – Bonferroni correction. Significant p-value is bolded.

Associations between selected variables were assessed using Spearman’s rank correlation. In exploratory analyses, generalized linear models were applied to examine relationships between serum apelin, myostatin, follistatin, and FSTL1 levels (dependent variables) and cardiovascular risk parameters (independent variables). Covariates were selected a priori based on their established relevance as cardiovascular risk factors. These regression analyses were considered hypothesis-generating; therefore, no correction for multiple testing was applied, and results should be interpreted with caution.

Missing data were minimal and handled with complete case analysis, which did not affect the overall results. The primary outcome was serum apelin concentration. Sample size estimation was based on expected changes in serum apelin concentration. Assuming α = 0.05 and 80% power to detect a significant between-group difference (with a mean level of 2000 units in the S group and 600 units in the K group, SD ≈ 1100), at least 18 participants per group were required. All analyses were two-tailed, and for the primary outcomes statistical significance was set at *p* < 0.05.

Detailed Materials and Methods are presented in Supplementary File 1.

## Results

**Fig. 1:**
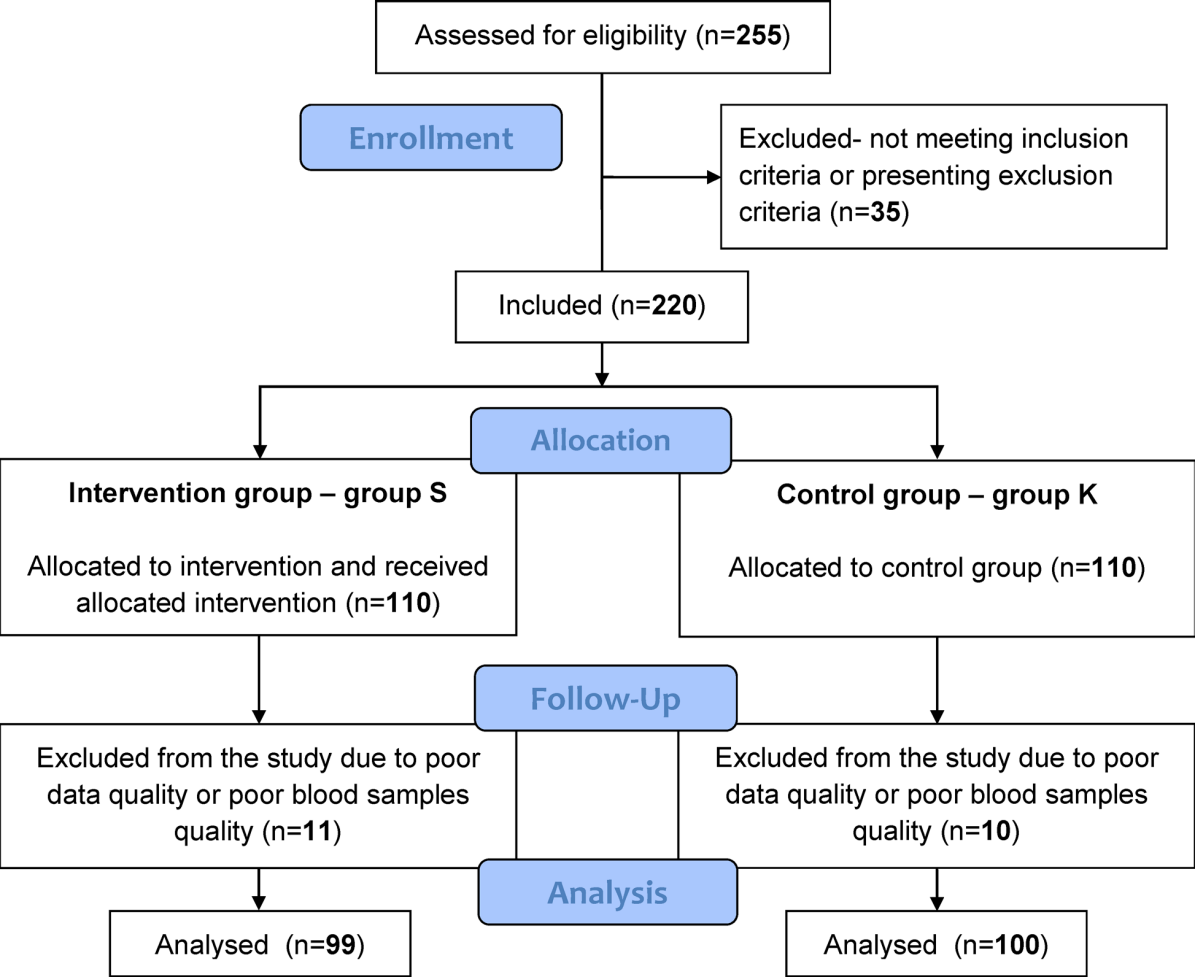
Flow diagram.

The study flow chart is shown in Fig. 1. We screened a random cohort of 255 patients who had experienced ACS. We excluded 35 because they did not meet the inclusion criteria or met one or more exclusion criteria. Thus, there were a total of 220 patients allocated to group S (*n* = 110) or group K (*n* = 110). All patients from group S underwent the study intervention. After completion of the study, we excluded 11 patients from group S and 10 patients from group K due to the poor of their data or blood samples. Hence, we considered a total of 199 patients for statistical analysis, 99 from group S and 100 from group K. There were no unintended effects or important harms. The study was finished after the last patient from group S had completed the cardiac rehabilitation programme and all data collection, measurement procedures and blood sample collection had ended.

The baseline characteristics of groups S and K are presented in Table 1. At baseline, body mass, BMI and systolic blood pressure were higher in group K compared with group S. Age, body height, diastolic blood pressure, heart rate and the serum apelin level did not differ between the groups at baseline. In addition, the sex distribution did not differ significantly between the groups (group S: 40 women and 59 men; group K: 39 women and 61 men; Fisher’s exact test, *p* = 0.8853).

At enrolment, the median post-ACS time was 4 weeks in group S and 22 weeks in group K (Mann–Whitney test, *p* < 0.0001). Supplementary Table 3 presents the characteristics of the CPX performed in group S – according to Bruce’s protocol or, in patients with relative contraindications to exercise testing according to ACC/AHA guidelines^35,36^, the 6MWT – to adjust the effort load during cardiac rehabilitation to each patient’s health state.

In the entire study population, according to available data, ACS was managed with the use of: percutaneous coronary intervention (PCI) with drug-eluting stent (DES) implantation in 87.6% of the patients; PCI and thrombectomy/thrombolysis in 3.4% of the patients; PCI with a drug-eluting balloon (DEB) in 1.4% of the patients; coronary artery bypass grafting (CABG) in 0.7% of the patients; PCI with CABG in 0.7% of the patients; plain old balloon angioplasty (POBA) in 4.1% of the patients; and a conservative treatment strategy in 2.1% patients.

In group S, the following pharmacotherapy was used: dual antiplatelet therapy (DAPT) in 84.5% of the patients; single antiplatelet therapy (SAPT) in 0% of the patients; DAPT and vitamin K antagonist (VKA)/non-VKA oral anticoagulants (NOAC) in 5.2% of the patients; SAPT and VKA/NOAC in 8.2% of the patients; NOAC only in 2.1% of the patients; beta blocker in 93.8% of the patients; angiotensin converting-enzyme inhibitors (ACEI) in 76.3% of the patients; angiotensin receptor blockers (ARB) in 17.5% of the patients; HMG-CoA (3-hydroxy-3-methylglutaryl coenzyme A) reductase inhibitors (statins) in 100% of the patients; ezetimibe in 12.4% of the patients; and fibrate in 6.2% of the patients. In group K, the following pharmacotherapy was used: DAPT in 57.7% of the patients; SAPT in 32.1% of the patients; DAPT and VKA/NOAC in 1.9% of the patients; SAPT and VKA/NOAC in 5.8% of the patients; NOAC only in 0% of the patients; beta blocker in 88.5% of the patients; ACEI in 67.3% of the patients; ARB in 19.2% of the patients; HMG-CoA reductase inhibitors (statins) in 96.2% of the patients; ezetimibe in 23.1% of the patients; fibrate 9,6% of the patients.

### Anthropometric and body composition

Table 2 presents the anthropometric and body composition analysis. In group S, the pre–post analysis revealed a higher body mass, BMI, and FFM after the intervention compared with baseline. On the contrary, MM and BMR were higher at baseline compared with after the intervention. Hip circumference, waist circumference, %FTC and FTC were comparable. In the between-groups analysis, BMI and waist circumference were higher in group K compared with group S, both at baseline and after the intervention, while body mass was higher in group K only compared to group S before intervention.

### Blood pressure and heart rate

Table 2 presents the blood pressure and heart rate results. Both systolic and diastolic blood pressure were lower in group S after the intervention compared with baseline; there were no intra-group differences in heart rate. Both systolic and diastolic blood pressure was lower in group S after the intervention compared group K.

### Biochemical results

Table 3 presents the detailed findings for the serum myokine levels. The serum apelin, myostatin and FSTL1 levels were higher in group S after the intervention compared with baseline; there was no change in follistatin. The serum apelin and myostatin levels were higher in group S after the intervention compared with group K. The serum follistatin level was higher in group K compared with group S at baseline and after the intervention, while the serum FSTL1 level was higher at baseline.

### Correlations and regression

Supplementary Table 4 provides details on the correlations between the serum myokine levels in group S. The serum follistatin level at baseline correlated positively with the serum myostatin level after the intervention and the serum follistatin level at baseline, and negatively with the serum apelin and FSTL1 levels at baseline. In addition, the serum myostatin level after the intervention correlated positively with the serum follistatin levels at baseline and after the intervention, and negatively with the serum apelin level at baseline. The serum apelin level after the intervention correlated negatively with the serum FSTL1 levels at baseline and after the intervention. The serum follistatin serum level at baseline correlated with the serum follistatin level after the intervention.

Supplementary Table 5 shows the correlations between the serum myokine levels in group K. The serum myostatin level correlated positively with the serum follistatin level and negatively with the serum FSTL1 and apelin levels. The serum apelin level correlated negatively with the serum follistatin level and positively with the serum FSTL1 level.

Supplementary Table 6 A–D present the linear regression analysis (y = β_1_x + β_0_) between the serum apelin, myostatin, follistatin and FSTL1 levels (y) and selected parameters of cardiovascular risk (heart rate, systolic blood pressure, diastolic blood pressure, body mass, BMI, %FTC, MM and MET in the exercise test). The serum apelin level in group S was independent of the cardiovascular risk factors at baseline and after the intervention. In group K, the serum apelin level was inversely related to systolic blood pressure. The serum myostatin level was independent of the cardiovascular risk factors in group S at baseline and after the intervention and in group K. The serum follistatin level was inversely related to diastolic blood pressure in group S after the intervention. In group S at baseline and in group K, the serum follistatin level was independent of the cardiovascular risk factors. The serum FSTL1 level was independent of the CV risk factors in group S at baseline and in group K. In group S, after the intervention the serum FSTL1 level was inversely related to %FTC and MM and directly related to body mass and BMI.

### Comparison of rehabilitated patients

The two most common forms of ACS are ST-segment elevation myocardial infarction (STEMI) and non-ST-segment elevation myocardial infarction (NSTEMI), which differ in pathophysiology, treatment and mortality^37^. In group S, there were 58 patients with STEMI and 35 patients with NSTEMI. Accordingly, we divided group S into STEMI and NSTEMI subgroups for further comparison. There was no difference in the sex distribution between the STEMI and NSTEMI subgroups (22 women and 36 men in the STEMI subgroup; 17 women and 18 men in the NSTEMI subgroup; Fisher’s exact test, *p* = 0.3870).

Table 4 shows comparisons of anthropometric and body composition parameters, blood pressure and heart rate between the two subgroups. In the NSTEMI subgroup, BMI was higher after the intervention compared with baseline, while MM, BMR, systolic blood pressure and diastolic blood pressure were lower after the intervention compared with baseline. At baseline, systolic blood pressure was higher in the NSTEMI subgroup compared with the STEMI subgroup.

Table 5 shows comparisons of the serum myokine levels between the two subgroups. The serum apelin and FSTL1 levels were higher after the intervention compared with baseline in the STEMI subgroup. The serum myostatin level was higher after the intervention compared with baseline only in the NSTEMI subgroup. The serum follistatin level did not differ between baseline and after the intervention in the STEMI or NSTEMI subgroup. Moreover, there were no differences in the serum myokine levels between the subgroups.

## Discussion

To the best of our knowledge, this study is the first to document the significant influence of cardiac rehabilitation on serum apelin, myostatin and FSTL1 levels in patients after ACS. Moreover, despite some significant correlations, we have shown that serum apelin, myostatin, follistatin and FSTL1 levels are independent of the classical cardiovascular risk factors in patients after ACS undergoing cardiac rehabilitation.

### Apelin

Apelin was first discovered as the orphan G protein-coupled receptor APJ ligand^20^. The gene that encodes apelin is located on chromosome Xq25-26.1^38^. In humans, apelin expression is high in the heart, endothelial cells, chondrocytes, brain, skin, spleen, lungs and thymus; intermediate in the skeletal muscle; and relatively low in the pancreas, liver and kidney^39^. There is high-quality published data on the beneficial role of apelin in cardiovascular system function. The following is a brief overview of the rich array of benefits that apelin provides for the circulatory system^17^. First, apelin decreases blood pressure and this effect is mediated mainly through nitric-oxide synthase (eNOS)^40^ as well as regulation of the protein kinase B (Akt)/eNOS pathway^41^. Elevated blood pressure is a well-proven cardiovascular risk factor leading to IHD, ACS, stroke and peripheral vascular diseases^42^, and the cardioprotective role of apelin mediated by its ability to lower blood pressure is indisputable. Second, apelin plays an invaluable cardioprotective role in conditions of hypoxia, especially myocardial ischaemia. In response to hypoxia, the expression of apelin and its receptor is elevated, along with activation of the phosphoinositide 3-kinase (PI3K)/Akt and extracellular signal-regulated kinase (ERK) pathways^43^, making apelin a crucial element of the hypoxia-counteracting system. During myocardial infarction, apelin decreases the infarct size and increases serum nitric oxide^44^. What is important in the context of our study is that apelin also significantly improves cardiac function and repair after myocardial infarction by increasing angiogenesis, stromal cell-derived factor-1α, chemokine receptor 4 (CXCR-4) and homing of vascular progenitor cells^45^. Apelin protects heart muscle against ischaemia/reperfusion injury by limiting the infarction size, ameliorating mechanical recovery or even reducing the ischaemia/reperfusion phenomenon itself^17,46^. Third, apelin improves cardiac function in conditions of heart failure frequently caused by ACS by increasing cardiac output and decreasing blood pressure and peripheral vascular resistance^47^. Finally, apelin acts against oxidative stress by diminishing ROS production and enhancing the activity of antioxidant enzymes.

We found that the serum apelin level almost doubled in group S after the intervention. Moreover, the serum apelin level was also significantly higher after the intervention in group S compared with group K. Hence, patients who do not undergo cardiac rehabilitation for many weeks after ACS have a low apelin level and they do not have a chance to benefit from the favourable effects of this myokine.

### Myostatin

Myostatin is a member of the TGF-β superfamily; it is encoded by the *MSTN* gene located on chromosome 2q32.2. Skeletal muscle presents the highest expression of myostatin, although fat tissue and heart muscle also show high expression^48^. Regarding the cardiovascular system, published data indicate that myostatin is an inhibitor of hyperplastic growth, cardiomyocyte proliferation and protein synthesis. Its increased expression in the heart is particularly a result of stress or developing pathology. In chronic heart failure, elevated myostatin production via the Smad-dependent pathway contributes to adverse cardiac remodelling. It leads to the reduction of cardiomyocyte hypertrophy and excessive synthesis of connective tissue^49^. In a similar way, myostatin impacts the heart in the context of myocardial infarction, whereas in animal model its absence is connected with decreased fibrosis and a higher survival rate^50^. Myostatin-mediated intensification of fibrosis also affects blood vessels, causing progressive vascular stiffness and the development of hypertension^51^. Another negative influence on the vascular wall is the inhibition of nitric oxide production in endothelial cells. The suggested mechanism includes downregulation of eNOS expression via the aforementioned Smad pathway^52^. Despite limited evidence, myostatin seems to have some crucial metabolic properties on the cardiovascular system. Tu et al.^53^ indicated that *MSTN* inactivation in mice has a protective impact on the development of insulin resistance, proatherogenic dyslipidaemia and atherosclerosis.

We observed a higher serum myostatin level in group S after the intervention compared with baseline and with group K. Given that previous studies have shown circulating myostatin is diminished in response to training^27,28^, our results require careful consideration. Saremi et al.^27^ evaluated healthy male subjects who received creatine supplementation and underwent resistance training, while Hittel et al.^28^ examined middle-aged men without cardiac disease and determined the myostatin level in muscles, but not in the blood. Therefore, our myostatin results may be the result of the myocardial pathology caused by ACS, which is well proven to upregulate myostatin^14,25,54^. A study by Casterillo et al. indicates that elevated myostatin levels can persist for up to 2 months following an ACS^55^. Another reason explaining the increase in myostatin is the positive correlation of its concentration with aerobic capacity, the improvement of which may be a result of rehabilitation^56^. However, the effect of elevated myostatin on the heart is ambiguous: on the one hand, it supports blood circulation in stress conditions^26^. It prevents metabolic alterations leading to the activation of glycolysis and glycogen accumulation, which may contribute to the development of heart failure^14^. On the other hand, in chronic states increased myostatin leads to unfavourable cardiac remodelling and fibrosis^49^. Nevertheless, considering that cardiac rehabilitation ameliorates cardiovascular system dysfunction after ACS, we hypothesise that the training implemented in our study represents an acute stressor on the heart, and elevated myostatin is a response to maintain proper circulation^26^. The exact role of myostatin in cardiac rehabilitation requires further investigation.

### Follistatin

Follistatin is a glycoprotein encoded by the *FST* gene located on chromosome 5q11.2 and expressed in almost all human tissues. Its general function is binding to and neutralisation of activin and other TGF-β family members^57^. As an inhibitor of myostatin, follistatin stimulates cardiac muscle growth and reduces fibrotic remodelling. In a rat model of myocardial infarction, the development of heart failure is associated with lower follistatin levels, indicating a positive impact on heart regeneration after injury^16^. Follistatin-dependent neutralisation of activin A specifically influences the vascular component of cardiac diseases. Activin A is a proinflammatory factor that promotes the expression of cell adhesion molecules, facilitating leucocyte adhesion and endothelial dysfunction. Impaired endothelial function leads to atherosclerotic lesions, whose building elements (macrophages, smooth muscle cells and endothelial cells) produce activin A to support disease progression^58,59^. On the other hand, elevated follistatin correlates with the incidence of type 2 diabetes. The underlying mechanism involves adipose tissue insulin resistance and diminished suppression of insulin-dependent lipolysis^60^.

We observed no significant changes in serum follistatin after cardiac rehabilitation. Considering that follistatin has antagonistic properties to myostatin^30^ and the fact that we observed increased serum myostatin in our patients after the intervention, we speculate that the effects of myostatin predominate over the effects of follistatin. As previously mentioned, increased myostatin expression in the heart is a result of a pathology such as ACS. The elevated level can persist for up to 2 months following the ischemic episode. The observed increase in myostatin alongside sustained follistatin concentration may be due to the stimulation of production via follistatin-independent pathways. Given the significant difference in the mean time since ACS in groups K and S (22 vs. 4 weeks), the elevated level of follistatin in group K may reflect the temporally delayed initiation of remodeling mechanisms that protect against the development of heart failure. This is supported by studies in animal models, which showed an increase in follistatin concentration at 4 and 8 weeks post-MI compared to the concentration noted after one week^61^. The presented results and considerations argue for the existence of more complex mechanisms regulating the expression of myostatin and follistatin and their mutual interactions, which require further investigation.

### FSTL1

FSTL1, produced by heart muscle cells^33^, promotes proliferation of stem cell–derived cardiomyocytes in conditions of hypoxia, so this myokine is likely one of the major factors responsible for efficient cardiac self-repair after ACS^18^. However, this still needs to be confirmed in human studies. In an animal model, FSTL1 is upregulated in the heart after myocardial infarction and ischaemia/reperfusion injury and protects cardiac myocytes against hypoxia/reoxygenation-induced apoptosis in a manner that is dependent on Akt and ERK^19^. Recently elevated FSTL1 levels have been proposed as a reliable early biomarker of cardiovascular risk in humans^62^, highlighting the increasingly recognised importance of this myokine in clinical cardiology and cardiological diagnostics.

We found a significant increase in the serum FSTL1 level in group S after the intervention, the first evidence that this myokine increases in response to cardiac rehabilitation in patients after ACS. On the other hand, we documented a higher serum FSTL1 level in group K compared with group S at baseline. Considering that FSTL1 may be a cardiovascular risk biomarker^62^, we hypothesise that cardiovascular risk in patients after ACS increases with time spent without cardiac rehabilitation and is notably high.

### Myokines: a link between cardiac rehabilitation and cardiovascular system function

Despite the undoubtedly advantageous effect of cardiac rehabilitation on the cardiovascular system in patients after ACS – rendering cardiac rehabilitation a highly recommendable procedure in this population^63,64^ – the pathways that link cardiac rehabilitation with cardiovascular benefits are not completely clear. Our study provides new details for this linkage.

Group S showed a reduction in systolic and diastolic blood pressure due to cardiac rehabilitation, consistent with previous studies documenting that even short-term cardiac rehabilitation significantly reduces blood pressure^65^. Apelin significantly decreases blood pressure^40,41^, and the serum apelin level increased after the intervention in our study. Thus, we presume that the hypotensive effect of cardiac rehabilitation in group S was partially mediated by apelin. Apelin also ameliorates cardiac function and repair after ACS via intensified angiogenesis and upregulation of stromal cell-derived factor-1α, CXCR-4 and homing of vascular progenitor cells^45^, and presents antioxidative properties^17^. Thus, we hypothesise that the beneficial effect of cardiac rehabilitation in patients after ACS is driven by an elevated serum apelin level.

Myostatin seems to exert rather unfavourable effects on the cardiovascular system. However, some studies have shown that myostatin stabilises the metabolic status and energy homeostasis of the heart and counteracts cardiac hypertrophy^14^. Therefore, we presume that this was the major role of elevated serum myostatin we observed after cardiac rehabilitation: heart muscle was not healthy and was negatively affected by ACS.

Although serum follistatin did not increase significantly after the intervention, we observed some negative correlations between the serum follistatin level and blood pressure in group S, which confirms the unquestionable cardioprotective effect of this myokine^15^. Considering the fact that the blood follistatin level increases during exercise^31,32^, we hypothesise that follistatin is also responsible for the benefits of cardiovascular system resulting from cardiac rehabilitation.

In a rat model of myocardial infarction, the blood FSTL1 level and FSTL1 expression in cardiac and skeletal muscles increase after aerobic training^34^. Recently, in an animal model of myocardial infarction, researchers reported that FSTL1 is a molecular link between 4 weeks of training and training-derived cardiovascular benefits. Specifically, FSTL1 expression is induced; elevated blood FSTL1 correlates positively with FSTL1 expression in skeletal muscles and correlates negatively with reduced cardiac fibrosis; functional performance improves; and angiogenesis is induced in the myocardium^66^. In group S, we documented a strong negative correlation between the serum FSTL1 level and maximum systolic blood pressure for the CPX according to Bruce’s protocol. Note that the maximum systolic blood pressure for the CPX is a well-proven cardiovascular risk factor for MACE^67^. Thus, we hypothesise that our 2-week cardiac rehabilitation protocol for patients after ACS increases serum FSTL1 and thus reduces the risk of MACE. This hypothesis needs to be tested in additional studies.

Our regression analysis of the relationship between serum myokine levels and cardiovascular risk parameters showed only a marginal association in case of FSTL1 and single associations for the other three myokines. This means that the favourable effect of myokines on the cardiovascular system in patients after ACS undergoing cardiac rehabilitation is not mediated by classical cardiovascular risk parameters (i.e. blood pressure, heart rate, body mass, BMI, FTC, MM and the result of the exercise test). Rather, these myokines, especially apelin, myostatin and follistatin, are independent factors linking cardiac rehabilitation with the resulting cardiovascular benefits. Our results support our hypothesis that myokines are not just another agent regulating cardiovascular system performance; they represent an independent pathway linking physical exercise with amelioration of cardiovascular system function of high cardioprotective potency in patients after ACS. While this hypothesis needs to be tested in future studies, if it is confirmed, it may bring numerous clinical benefits for patients with cardiovascular diseases. Nevertheless, based on our results and the literature, serum myokine levels should be interpreted as a new generation of biomarkers of cardiac rehabilitation efficiency in patients after ACS.

### Comparison between patients with STEMI and NSTEMI

For group S, when we compared the STEMI and NSTEMI subgroups, we saw no differences in myokine levels at baseline or after the intervention. Furthermore, pre–post comparisons for the serum follistatin levels were the same for the STEMI and NSTEMI subgroups. After performing the Bonferroni correction for apelin, statistical significance in the pre-post comparison was maintained exclusively in the STEMI group. The observed difference may be the result of its cardioprotective effect when exposed to hypoxia and the reduction of the ischemia/reperfusion phenomenon, the severity of which is greater in STEMI patients. However, the serum myostatin level increased only in the NSTEMI subgroup, while the serum FSTL1 level increased only in the STEMI subgroup, in which it more than doubled. These results strongly suggest that these two types of ACS differ significantly not only in the pathophysiology, but also in the response to cardiac rehabilitation. As myostatin improves the metabolic status, energy homeostasis and proper circulation in the heart and prevents cardiac hypertrophy^14,26^, our results indicate that only patients with NSTEMI experience these benefits of myostatin in response to cardiac rehabilitation. The lack of such a myostatin response in patients with STEMI emphasises the extreme cardiac pathology of STEMI. Conversely, the serum FSTL1 level increased in response to cardiac rehabilitation only in patients with STEMI. This suggests that this group of patients could benefit more from the effect of this myokine. However, the serum FSTL1 level did not differ between the STEMI and NSTEMI subgroups after the intervention. This suggests that patients with STEMI had a relatively low serum FSTL1 level at baseline, again highlighting the more serious cardiac pathology of STEMI compared with NSTEMI.

### Future scientific perspective

The issue of myokine response to cardiac rehabilitation in patients after ACS has not been well investigated. Thus, many issues require further clarification. Currently, the influence of different training modalities on myokine response remains unknown. Moreover, it should be clarified how the duration of each training session and the entire training programme alter serum myokine levels. Finally, it would be reasonable to investigate the issue of myokines at the molecular level, especially how physical training modifies the expression of different myokines in cardiac muscle. Future research on this topic should be multi-center and focus on a long-term assessment that includes changes in myokine concentrations, the factors influencing them, but also clinical aspects such as the effect of cardiac rehabilitation on exercise tolerance and survival. Further investigation is also warranted for the gaps in foundational knowledge concerning myokines demonstrated in this study - namely, the impact of the superimposition of ACS and physical exercise on myostatin concentration changes, and the precise action of the follistatin-myostatin axis in this specific context.

### Clinical implications

Our results have numerous clinical implications. First, we showed that a 2-week cardiac rehabilitation protocol is long enough to induce beneficial myokine response and that it is not too short even for clinical use. Second, based on ours and others’ outcomes, in the future cardiac rehabilitation training protocols may be modified to yield the most favourable myokine response for the patient after ACS. Third, we demonstrated that patients with STEMI and NSTEMI differ in their myokine response after cardiac rehabilitation. Thus, it seems that future cardiac rehabilitation protocols should be tailored for different types of patients after ACS and individualised regarding the training modality and duration to maximise the clinical cardiovascular benefits for the patient. Fourth, determination of serum myokine levels should be considered for clinical use to stratify a patient’s cardiovascular risk and to predict a patient’s response to cardiac rehabilitation. The results regarding changes in myokine concentrations and clinical cardiovascular parameters in the study group showed parallel trends in both sets of measures—specifically, an increase in apelin, myostatin, and FSTL1 accompanied by a decrease in SBP and DBP. However, subsequent logistic regression analysis revealed no significant correlation between these variables. Therefore, the identified myokines should be regarded as independent indicators of favorable physiological adaptation to exercise. Finally, based on ours and others’ studies, in the near future supplementation with exogenous myokines may be investigated and implemented for patients after ACS to increase the advantageous effect of cardiac rehabilitation or even to enable to obtain such an effect in patients who may be unable to perform physical exercise.

### Study strengths

The major strength of our study is its novelty: to the best of our knowledge, it is the first trial investigating the myokine response to cardiac rehabilitation in patients after ACS. Furthermore, our results should be translatable into future clinical guidance for cardiac rehabilitation. We investigated several outcomes – the serum levels of four myokines as well as anthropometric and cardiovascular parameters – which enabled us to compare the patients’ responses to cardiac rehabilitation and to situate our results in the broader clinical context. We also compared the myokine response between patients with STEMI and NSTEMI to provide new knowledge on the differences between these two ACS types. Finally, we enrolled a relatively high number of patients in our trial, which strongly increased the credibility of our outcomes. This study is a part of the CARDIO-REH randomised study and represents an extension and continuation of our previous research^65^.

### Study limitations

The greatest limitation of our study is its relatively short duration. However, even a 2-week cardiac rehabilitation protocol yielded significant changes in the myokine response, and these findings have clinical implications for the future treatments of patients recovering from ACS. A further limitation of the short observation period is the limited ability to determine the clinical impact of cardiac rehabilitation, specifically through a comparison of exercise tolerance, readmission rates, and long-term survival nevertheless, it is important to emphasize that the primary objective of the study was to evaluate only the short-term effect of cardiac rehabilitation. Due to the aforementioned short observation period, the long-term effect on myokine concentration is unclear and provides a basis for planning further research in this direction. Second, the study population was recruited in a single center, thus a study group is relatively homogenous. The participants were similar in age, clinical status, and pharmacological therapy, which strengthens the internal validity of our observations but may compromise their external validity. This homogeneity also extended to a general lack of advanced comorbidities or severe functional limitations. For this reason, our results may not be representative of outcomes in older, frailer patients or those with significant conditions like chronic heart failure, renal disease, or morbid obesity. Although women constituted 36–40% of the study group – the sample size was insufficient to draw robust conclusions regarding sex-specific responses to CR. On the other hand, the study was conducted in an inpatient cardiac rehabilitation medical center rather than in an outpatient setting, which ensured appropriate adherence to the rehabilitation program and measurements standardization. Third, we did not examine myokine gene expression; we only used ELISA to measure serum levels. In a human study it would be necessary to obtain tissue biopsies to measure gene expression, a procedure that would have been quite harmful for our patients, especially considering that the great majority of them, in accordance with medical guidance, used antiplatelet therapy and thus have an increased risk of bleeding. Moreover, the use of ELISA allowed us to include a relatively large number of patients, which enhanced the reliability and value of our results. The baseline difference between the study and control groups in the post-ACS period also constitutes a limitation of our study. However, this difference arises from the fact that patients in group S were recruited during the recommended period for cardiac rehabilitation implementation, whereas patients in group K did not undergo cardiac rehabilitation. Therefore, this limitation should be taken into account and the results interpreted with caution.

Although the sample size was determined a priori based on expected differences in apelin levels, the relatively small number of participants still limits the generalizability of the findings. Moreover, the study may have been underpowered to detect smaller effect sizes for other biochemical outcomes, which increases the risk of type II error. We also acknowledge that the sample size was calculated based on the primary outcome and is not sufficient to conduct adequately powered subgroup (STEMI/NSTEMI) analyses. Thus, subgroup analyses were exploratory and should be interpreted with caution. Another limitation concerns the issue of multiple comparisons. While Bonferroni correction was applied to comparative statistics, the correlation and regression analyses were exploratory and thus not corrected for multiple testing. This approach increases the risk of type I error, and the results should be considered as preliminary, requiring further replication.

## Conclusions

In patients after ACS, a 2-week cardiac rehabilitation increased the serum apelin, myostatin and FSTL1 levels. These three myokines seem to be independent factors linking cardiac rehabilitation with its cardiovascular benefits. Patients with STEMI and NSTEMI differed in their myokine response to cardiac rehabilitation, especially in the case of apelin, myostatin and FSTL1. The associations between cardiac rehabilitation and myokines requires further scientific investigation.

Author contributions: Conceptualization: Damian Skrypnik. Data curation: Damian Skrypnik. Formal analysis: Damian Skrypnik. Funding acquisition: Damian Skrypnik. Investigation: Damian Skrypnik, Dawid Woszczyk and Katarzyna Skrypnik.

Methodology: Damian Skrypnik, Katarzyna Skrypnik and Joanna Suliburska.

Project administration: Damian Skrypnik.

Resources: Damian Skrypnik, Katarzyna Skrypnik and Joanna Suliburska.

Software: Damian Skrypnik, Katarzyna Skrypnik, José Casaña Granell, and Joanna Suliburska.

Supervision: Damian Skrypnik.

Validation: Damian Skrypnik, Katarzyna Skrypnik, José Casaña Granell, and Joanna Suliburska.

Visualization: Damian Skrypnik, José Casaña Granell, and Dawid Woszczyk.

Writing–original draft: Damian Skrypnik and Dawid Woszczyk (Dawid Woszczyk prepared some parts of the Discussion section).;

Writing–review and editing: Katarzyna Skrypnik, José Casaña Granell, and Joanna Suliburska.

All authors have read and agreed to the published version of the manuscript.

## Supplementary Information

Below is the link to the electronic supplementary material.

Supplementary Material 1

Supplementary Material 2

Supplementary Material 3

Supplementary Material 4

Supplementary Material 5

Supplementary Material 6

Supplementary Material 7

Supplementary Material 8

Supplementary Material 9

Supplementary Material 10

## Data Availability

The data that support the findings of this study are available from the corresponding author upon reasonable request.
