# Supplementary material for: Cardiac rehabilitation influences serum myokine levels in patients after acute coronary syndrome: the randomised CARDIO-REH study
Source: Sci Rep. 2025 Nov 6;15:38951. doi: 10.1038/s41598-025-22897-0 (PMC12592514; doi:10.1038/s41598-025-22897-0)
Supplement: Supplementary file 1 — Supplementary Material 1 [file 41598_2025_22897_MOESM1_ESM.pdf]

Title: Cardiac rehabilitation influences serum myokine levels in patients after acute coronary syndrome: the randomised CARDIO-REH study
Authors: Damian Skrypnik; Katarzyna Skrypnik; José Casaña Granell; Dawid Woszczyk; Joanna Suliburska
Scientific Reports

MATERIALS AND METHODS

Study design

The trial was designed as a randomised, interventional, prospective and comparative study. The study protocol was approved by the Bioethics Committee, Poznan University of Medical Sciences (no. 476/19 with amendments). The project followed the Declaration of Helsinki (1975 revision with amendments). The study was conducted in Poznan University of Medical Sciences, Poznan, Poland. The study lasted from April 2019 to September 2023. The CONSORT recommendations were implemented. The trial has been registered at ClinicalTrials.gov under NCT03935438 (the first registration date 02/05/2019; https://clinicaltrials.gov/study/NCT03935438?term=NCT03935438&rank=1).

Patients meeting all inclusion criteria and not presenting any of exclusion criteria were enrolled and divided into two groups: a study group (group S) and a control group (group K) with an allocation ratio of 1:1. Patients who had ACS 2–9 weeks before the enrolment were included in group S. After assignment, patients from group S were submitted to a 2-week cardiac rehabilitation programme, which was the study intervention. Patients who had ACS within the period of not less than 9 weeks prior to enrolment and had not yet been subjected to cardiac rehabilitation were enrolled into group K. In patients from group S, anthropometric, blood pressure, heart rate and body composition measurements were taken at baseline and after the 2-week intervention, along with blood sampling. The same measurements and blood sample collection as in patients from group S were performed in patients from group K, but only once. In all patients from group S, the cardiac stress test (CPX) was performed before the intervention. As recommended by the Polish Cardiac Society (PCS) [9], which is a member of the ESC, the CPX was necessary to determine each patient's cardiovascular risk and exercise tolerance. This information was indispensable to adjust the patient's effort load during cardiac rehabilitation to their health state. After enrolment, the patient's electrocardiographic (ECG) records and medical documentation (including data on ACS pharmacotherapy and management and diabetes) were analysed by the physician. To detect previously unregistered symptoms of cardiac ischaemia or arrhythmias constituting contraindication to exercise, all

patients underwent 12-lead ECG immediately after enrolment. Medical care was provided to all patients during the entire study. No important changes to the methods were introduced after trial commencement. After the trial commenced, there were no changes to the study outcomes. No interim analyses were performed. This study is a part of the CARDIO-REH randomised study and represents an extension and continuation of our previous research [65].

Study patients

All patients gave their written informed consent to participate in the trial. The inclusion criteria were: females and males aged ≥ 18 years who signed an informed consent form and had experienced ACS 2–9 weeks prior to inclusion (group S) or within the period of not less than 9 weeks prior to inclusion without cardiac rehabilitation at the time of enrolment (group K). The exclusion criteria were: a clinically significant chronic or acute inflammatory process in the digestive, genitourinary or respiratory tract or in the throat, paranasal sinuses or mouth; connective tissue disease; previous cardiac rehabilitation (phase I cardiac rehabilitation [35] due to PCS recommendations [9] was allowed in patients in both groups); alcohol or drug abuse; active neoplastic disease; lactation; pregnancy; or any other disorders that in the opinion of the investigators could pose a risk to the patient's health during the trial or limit the effectiveness and credibility of the trial. Patients with absolute contraindications to exercise testing in the line with American College of Cardiology/American Heart Association (ACC/AHA) guidelines were not included in the trial. These contraindications were: acute myocardial infarction within 2 days; high-risk unstable angina; active endocarditis; uncontrolled cardiac arrhythmias leading to symptoms of hemodynamic compromise; symptomatic and uncontrolled heart failure; pulmonary infarction; acute pulmonary embolus; symptomatic severe aortic stenosis; acute aortic dissection; acute myocarditis or pericarditis; physical disability precluding safe and adequate test performance; acute noncardiac disease affecting exercise performance or that may be aggravated by exercise (e.g. renal failure, infection or thyrotoxicosis); and an inability to provide written informed consent [35,36]. Patients who met all of the inclusion criteria and did not have any of the exclusion criteria were enrolled in the trial. During the trial, the occurrence of any of exclusion criterion resulted in withdrawal of the patient from the study. Patient age and sex were self-reported.

Anthropometric parameters and body composition measurement

Anthropometric measurements and body composition analyses were measured in the morning (with the patient wearing light clothing and no shoes) after the patient had fasted overnight.

Body mass was measured with an electronic scale (model 370, InBody Co., Seoul, Korea) to the nearest 0.1 kg and height was measured with a manual stadiometer to the nearest 0.1 cm. Body mass index (BMI) was calculated as the body mass divided by the height squared (kg/m^2). The waist circumference was measured in the horizontal plane midway between the iliac crest and the lowest rib at the end of normal expiration. The hip circumference was measured at the greatest protuberance of the buttocks. The waist and hip circumferences were determined in standing position with non-stretchable tape to the nearest 0.5 cm.

Electrical bioimpedance was measured with the same piece of equipment mentioned above (model 370, InBody Co.) for body composition analysis. Before the procedure, the patient cleaned their feet and hands with antibacterial solution. The body composition measurement took approximately 1–1.5 min. The patient's gender, age and height were recorded on the device. Subsequently, the patient assumed an upright motionless position on the device with their hands grabbing the upper electrodes and their feet centred on the lower electrodes. Their arms were set wide to prevent contact with the torso. After the analysis, the patient was told to step down from the device. The mass of fat tissue content (FTC), the percentage fat tissue content (%FTC), muscle mass (MM), fat-free mass (FFM) and the basal metabolic rate (BMR) were registered.

Blood pressure measurement

Blood pressure was determined with the use of a digital electronic tensiometer (model 705IT TM, Omron Corporation, Kyoto, Japan). Physical exercise and caffeine consumption for at least 30 minutes before measurement were not allowed. During the measurement, the patient sat in a chair with their feet on the floor and back supported for > 5 min, with an empty bladder and after relaxation. Standard or large-size cuffs for adults were utilised. Blood pressure was determined in both the right and left arms; the higher blood pressure was recorded, and that arm was used for the subsequent measurements. Three additional measurements were recorded and the mean was calculated. During the blood pressure determination, the patient's arm rested on a table. Heart rate was determined under the same conditions by using a stethoscope for heart auscultation.

Blood sample collection and biochemical analysis

Blood samples were collected at room temperature in the morning after the patient had fasted and had a full-night's sleep. Prior caffeine intake was not allowed. Before sample collection, the patient was in the supine for 30 min in silence. Blood samples were collected from the

ulnar vein into serum-separator tubes. After preparation, serum samples were immediately frozen and stored at −80 °C. Serum myostatin (GDF8), follistatin, apelin and FSTL1 levels were determined with commercially available enzyme-linked immunosorbent assay (ELISA) kits (RayBiotech, Peachtree Corners, GA, USA). An Infinite F50 spectrometer (Tecan Group Ltd., Männedorf, Switzerland) was used for spectrometry.

CPX- cardiac stress test

Before the cardiac rehabilitation programme commenced, all patients from group S were subjected to a CPX to adjust the effort load during cardiac rehabilitation to the patient's health state. The basic CPX was performed according to Bruce's protocol. Patients with relative contraindications to exercise testing in the line with ACC/AHA guidelines performed the 6-Minute Walk Test (6MWT) rather than the CPX. These relative contraindications were: left main coronary stenosis or its equivalent, moderate stenotic valvular heart disease, tachyarrhythmia or bradyarrhythmia, severe arterial hypertension, atrial fibrillation with an uncontrolled ventricular rate, electrolyte abnormalities, hypertrophic cardiomyopathy and other forms of outflow tract obstruction, high-degree atrioventricular block, and mental impairment leading to an inability to cooperate [35,36].

The patients were instructed not to smoke or eat for 3 h before the CPX and not to perform intense physical exercise for at least 12 h before testing [35,36]. The CPX was performed in an exercise laboratory, under a physician's supervision, between 8:00 a.m. and 10:00 a.m., and according to the Bruce's protocol in the line with the AHA guidelines [35,36]. The test was performed on a treadmill (Aspel B612 model C, Aspel S.A., Zabierzów, Poland). Before commencing, the standing blood pressure was measured and a resting standard 12-lead ECG was performed. Bruce's protocol comprises seven stages, in which the exercise load increases every 3 min due to an increase in the treadmill velocity and slope angle. The treadmill velocity and percent grade were 1.7 mph and 10%, respectively, for the first stage; 2.5 mph and 12%, respectively, for the second stage; 3.4 mph and 14%, respectively, for the third stage; 4.2 mph and 16%, respectively, for the fourth stage; 5.0 mph and 18%, respectively, for the fifth stage; 5.5 mph and 20%, respectively, for the sixth stage; and 6.0 mph and 22%, respectively, for the seventh stage. During the test, blood pressure was measured every 3 min, and continuous ECG was performed. The test was completed when the pulse limit was reached or when there were contraindications to continue based on the AHA recommendations: moderate-to-severe angina; ST-segment elevation (> 1.0 mm) in leads without Q waves (other than V1 or aVR); central nervous system symptoms (e.g. dizziness,

ataxia or near syncope); a > 10 mmHg decrease in systolic blood pressure (persistently below baseline); sustained ventricular tachycardia; signs of poor perfusion (pallor or cyanosis); ST or QRS changes such as excessive ST displacement (horizontal or down-sloping of > 2 mm) or a marked axis shift; increasing chest pain or wheezing, leg cramps, shortness of breath, claudication or fatigue; technical difficulties in ECG and/or blood pressure monitoring; patient's request to stop; arrhythmias other than sustained ventricular tachycardia; a hypertensive response (systolic blood pressure > 250 mmHg and/or diastolic blood pressure > 115 mmHg); and the development of bundle-branch block that cannot be distinguished from ventricular tachycardia [35,36]. The pulse limit at which the CPX was completed was calculated based on the AHA recommendations as 70% of the age-predicted maximum heart rate. The age-predicted maximum heart rate was calculated with the use of the formula 220 – age in years [36]. A recovery phase was included in the observation, during which continuous ECG was performed and blood pressure was measured. The maximum heartrate (HRmax), maximum blood pressure (BPmax), total exercise duration and metabolic equivalents of the task (MET) were measured, as per Bruce's protocol.

Due to the AHA guidelines [36], in subjects from group S with relative contraindications to exercise testing according to the ACC/AHA guidelines [35,36], or with peripheral arterial occlusive disease or marked left ventricle dysfunction who were unable to perform CPX in the line with Bruce's protocol, the 6MWT was performed [36]. Directly prior to 6MWT, BP, heart rate, and blood oxygen saturation (SO2) measurements were taken and a 12-lead ECG was done. Patients were instructed to walk down a thirty-metre corridor and attempt to cover as much ground as possible in 6 min at their own pace. At the completion of the six-minute period, the patients stopped walking, the total distance walked was measured and the symptoms reported by the patient were recorded. Directly after walk discontinuation, HRmax; BPmax; and SO2 measurements were taken. BP, HR and SO2 were also determined 1 min and 2 min after the walk discontinuation [36]. After the end of the 6MWT, the 12-lead ECG was undertaken. Mean walk velocity during the test was determined. MET was calculated due to the formula MET = $[0.1 \times \text{velocity } (\text{m}\cdot\text{min}^{-1}) + 3.5\text{mLO}_2\cdot\text{kg}\cdot\text{min}^{-1}] \div 3.5\text{mLO}_2\cdot\text{kg min}^{-1}$ [35,36].

Intervention

The patients in group S underwent the cardiac rehabilitation programme [35] in a cardiac rehabilitation medical centre in the line with PCS guidelines [9]. The intervention lasted 2 weeks. The patients were qualified for the programme by a physician based on their medical

history and the CPX results. The programme was adjusted to each patient's exercise tolerance and health state according to the PCS recommendations [9]. The rehabilitation programme was conducted in the cardiac rehabilitation room by a trained physiotherapist under medical supervision. The patient's blood pressure and heart rate were measured before, during and after each training set.

The cardiac rehabilitation programme consisted of continuous and interval training on a cycle ergometer (CRG 200, Aspel) accompanied by continuous ECG monitoring (Aster Beta System XL, Aspel), free active exercises, isokinetic exercises, isotonic exercises, isometric exercises, individual and group general fitness exercises, active breathing exercises with and without resistance, balance exercises, active resistance exercises, marching training with and without equipment, walking, and station training. The programme was performed in small groups of 2–4 patients or individually. The training was performed daily for a total of 14 days, with no breaks. There were three training sets each day, with a total duration of 30–90 min per day, depending on the patient's exercise tolerance and health, in the line with PCS recommendations [9]. The exercise sets began at 8:00 a.m., 10:30 a.m. and 01:00 p.m. Each set started with 5–10 min of aerobic warm-up followed by the main cardiac rehabilitation training. The rehabilitation set ended with 5–10 min of stretching, breathing and cool-down aerobic exercises. According to the PCS guidelines [9], the main cardiac rehabilitation training was executed in the line of one of four models (A, B, C or D), depending on the patient's CPX results and cardiovascular risk grade (with each patient allocated by a physician) (see **Supplementary Table 1**). Heart rate reserve (HRR) was defined due to PCS guidelines with the formula: HRR = maximum heart rate during CPX (HRmax) – resting heart rate (HRrest) [9]. **Supplementary Table 2** shows the patients' cardiovascular risk grades in the line with PCS statement guidelines [9]. To increase patient safety, a cardiac defibrillator, an ECG monitor, resuscitation medication and equipment were available during training. The resuscitation crew consisting of an anaesthesiologist and medical rescuer could be summoned immediately. Except for the cardiac rehabilitation programme undertaken by group S, there were no differences in the study procedures between group S and group K.

Patient allocation and statistical analysis

All included patients were assigned a unique code by an independent member of the study team. This team member maintained a secure database connecting codes with subject identifiers but was not involved in any further study procedures. The random allocation sequence was computer-generated using permuted blocks of variable size. Randomisation was

stratified according to the time since ACS: subjects within 2–9 weeks after ACS were allocated to the cardiac rehabilitation intervention arm (group S), while patients who experienced ACS within the period of not less than 9 weeks were allocated to the control arm (group K). Within each stratum, patients were randomly assigned in a 1:1 ratio. No additional stratification (e.g. by STEMI/NSTEMI status or other baseline characteristics) was implemented.

The randomisation list contained only study codes, with no patient identifiers, and was provided exclusively to the study physician responsible for group assignment. Allocation concealment was ensured by restricting access to the randomisation list to this physician only. Researchers involved in data collection, outcome assessment, and statistical analysis were blinded to group allocation throughout the study. Patients were not blinded due to the nature of the study.

Statistical analyses were performed using Statistica version 13 (StatSoft, Tulsa, OK, USA). Data distribution was assessed with the Shapiro–Wilk test. Continuous variables are presented as median [Q1- first quartile; Q3- third quartile]. Baseline characteristics of the study and control groups (Table 1) are reported descriptively; these comparisons were not adjusted for multiple testing, as they were intended to describe the sample rather than to test study hypotheses. For the main analyses (Table 2, Table 3, Table 4, Table 5), within-group changes before and after cardiac rehabilitation were compared using paired Student's t-tests or Wilcoxon signed-rank tests, depending on normality. Between-group comparisons were performed with independent Student's t-tests or Mann–Whitney U tests. Fisher's exact test was used for categorical variables. To control for multiple testing in the main outcomes, p-values were adjusted using the Bonferroni procedure.

Associations between selected variables were assessed using Spearman's rank correlation. In exploratory analyses, generalized linear models were applied to examine relationships between serum apelin, myostatin, follistatin, and FSTL1 levels (dependent variables) and cardiovascular risk parameters (independent variables). Covariates were selected a priori based on their established relevance as cardiovascular risk factors. These regression analyses were considered hypothesis-generating; therefore, no correction for multiple testing was applied, and results should be interpreted with caution.

Missing data were minimal and handled with complete case analysis, which did not affect the overall results. The primary outcome was serum apelin concentration. Sample size estimation was based on expected changes in serum apelin concentration. Assuming $\alpha = 0.05$ and 80% power to detect a significant between-group difference (with a mean level of 2000

units in the S group and 600 units in the K group, SD ≈ 1100), at least 18 participants per group were required. All analyses were two-tailed, and for the primary outcomes statistical significance was set at $p < 0.05$.
