# Supplementary material for: Cardiac rehabilitation influences serum myokine levels in patients after acute coronary syndrome: the randomised CARDIO-REH study
Source: Sci Rep. 2025 Nov 6;15:38951. doi: 10.1038/s41598-025-22897-0 (PMC12592514; doi:10.1038/s41598-025-22897-0)
Supplement: Supplementary file 2 — Supplementary Material 2 [file 41598_2025_22897_MOESM2_ESM.pdf]

Title: Cardiac rehabilitation influences serum myokine levels in patients after acute coronary syndrome: the randomised CARDIO-REH study

Authors: Damian Skrypnik; Katarzyna Skrypnik; José Casaña Granell; Dawid Woszczyk; Joanna Suliburska

Scientific Reports

Supplementary Table 1. Models of main cardiac rehabilitation training according to Polish Cardiac Society guidelines [9]

Model	CV risk	CPX result – exercise tolerance	Total daily training duration [min]	Training intensity
**A**	low	≥ 7 MET	60 – 90	60 – 80 % HRR or 50 – 70 % HRmax
**B**	mid	≥ 5 MET	45 – 60	50 – 60 % HRR or 50 % HRmax
**C**	mid	3 – 5 MET	45	40 – 50 % HRR or 40 – 50 % HRmax
	high	≥ 6 MET		
**D**	mid	< 3 MET	30 – 45	< 20 % HRR or < 110 – 115 % HRrest
	high	< 6 MET		

CPX: cardiac stress test; CV: cardiovascular; HRmax: maximum heart rate during CPX; HRrest: resting heart rate; HRR: heart rate reserve; MET: metabolic equivalent of task; min: minutes. HRR = maximum heart rate during CPX – resting heart rate [9]
