# Supplementary material for: Cardiac rehabilitation influences serum myokine levels in patients after acute coronary syndrome: the randomised CARDIO-REH study
Source: Sci Rep. 2025 Nov 6;15:38951. doi: 10.1038/s41598-025-22897-0 (PMC12592514; doi:10.1038/s41598-025-22897-0)
Supplement: Supplementary file 3 — Supplementary Material 3 [file 41598_2025_22897_MOESM3_ESM.pdf]

Title: Cardiac rehabilitation influences serum myokine levels in patients after acute coronary syndrome: the randomised CARDIO-REH study
Authors: Damian Skrypnik; Katarzyna Skrypnik; José Casaña Granell; Dawid Woszczyk; Joanna Suliburska
Scientific Reports

Supplementary Table 2. Patient's cardiovascular risk grade stratification according to Polish Cardiac Society guidelines [9]

**Parameter**	**Cardiovascular risk grade**		
	**Low***	**Mid****	**High****
LVEF	≥ 50%	36-49%	≤35%
Complex ventricular arrhythmia	Absent at rest and during exercise		Present at rest and during exercise
Symptoms of ischemia on an exercise ECG	Absent	ST segment depression ≥ 1 mm and ≤ 2 mm	ST segment depression > 2 mm
Exercise tolerance based on CPX	≥ 7 MET	5–6.9 MET	< 5 MET
Hemodynamic reaction to exercise during CPX	normal		No increase or decrease in SBP or HR with increasing exercise load
Clinical data	Uncomplicated MI, CABG, PCI		MI or cardiac intervention (e.g. PCI, CABG) complicated by shock; heart failure; recurrences of ischemia after invasive treatment

CABG: coronary artery bypass grafting; CPX: cardiac stress test; ECG: electrocardiogram; HR: heart rate; LVEF: left ventricular ejection fraction (the most current data based on medical documentation was used); MET: metabolic equivalent of task; MI: myocardial infarction; PCI: percutaneous coronary intervention; SBP: systolic blood pressure [9]

* all criteria present; ** at least one criterion present
