# Supplementary material for: Cardiac rehabilitation influences serum myokine levels in patients after acute coronary syndrome: the randomised CARDIO-REH study
Source: Sci Rep. 2025 Nov 6;15:38951. doi: 10.1038/s41598-025-22897-0 (PMC12592514; doi:10.1038/s41598-025-22897-0)
Supplement: Supplementary file 4 — Supplementary Material 4 [file 41598_2025_22897_MOESM4_ESM.pdf]

Title: Cardiac rehabilitation influences serum myokine levels in patients after acute coronary syndrome: the randomised CARDIO-REH study

Authors: Damian Skrypnik; Katarzyna Skrypnik; José Casaña Granell; Dawid Woszczyk; Joanna Suliburska

Scientific Reports

Supplementary Table 3. Baseline characteristics of group S – CPX results

**Bruce's protocol**		**6 minutes walk test**	
**Parameter**	**Value**	**Parameter**	**Value**
n	19	n	80
Total exercise duration [s]	482 [361; 593]	HRmax [bpm]	79 [69; 88]
HRmax [bpm]	135 [122; 144]	SBPmax [mmHg]	133 [117; 148]
SBPmax [mmHg]	145 [130; 155]	DBPmax [mmHg]	75 [64; 80]
DBPmax [mmHg]	85 [75; 90]	SO2 [%]	97 [96; 99]
Metabolic equivalent of task [MET]	7.4 [4.4; 10.4]	Total distance walked [m]	390 [315; 480]
		Mean walk velocity [m/min]	65.0 [52.5; 80.0]
		Metabolic equivalent of task [MET]	2.86 [2.50; 3.29]

Data are presented as median [Q1; Q3]. bpm, beats per minute; CPX, cardiac stress test; DBPmax, maximum diastolic blood pressure; HRmax, maximum heart rate; MET, metabolic equivalent of task; Q1: first quartile; Q3: third quartile; SBPmax, maximum systolic blood pressure; SO2, blood oxygen saturation at the completion of 6 minutes walk test
