# Supplementary material for: Cardiac rehabilitation influences serum myokine levels in patients after acute coronary syndrome: the randomised CARDIO-REH study
Source: Sci Rep. 2025 Nov 6;15:38951. doi: 10.1038/s41598-025-22897-0 (PMC12592514; doi:10.1038/s41598-025-22897-0)
Supplement: Supplementary file 5 — Supplementary Material 5 [file 41598_2025_22897_MOESM5_ESM.pdf]

Title: Cardiac rehabilitation influences serum myokine levels in patients after acute coronary syndrome: the randomised CARDIO-REH study

Authors: Damian Skrypnik; Katarzyna Skrypnik; José Casaña Granell; Dawid Woszczyk; Joanna Suliburska

Scientific Reports

Supplementary Table 4. Significant correlations between registered parameters in group S

Correlations between biochemical parameters					
GDF 8 (I) & GDF 8 (II)	0.77	GDF 8 (II) & Follistatin (I)	0.34	FSTL1 (I) & Apelin (II)	-0.28
GDF 8 (I) & Follistatin (I)	0.20	GDF 8 (II) & Follistatin (II)	0.21	FSTL1 (II) & Apelin (II)	-0.26
GDF 8 (I) & FSTL1 (I)	-0.29	GDF 8 (II) & Apelin (I)	-0.37		
GDF 8 (I) & Apelin (I)	-0.44	Follistatin (I) & Follistatin (II)	0.64		
**Correlations between biochemical parameters and anthropometric, body composition parameters, BP, HR, CPX/6MWT results**					
Follistatin (I) & age	0.22	Follistatin (I) & SBP (II)	-0.25	Follistatin (I) & 6MWT mean walk velocity	-0.25
Apelin (I) & age	0.29	Follistatin (II) & SBP (II)	-0.38	Follistatin (I) & 6MWT metabolic equivalent of task	-0.25
Apelin (I) & BMI (II)	0.30	Follistatin (I) & DBP (II)	-0.25	GDF 8 (II) & B-CPX DBPmax	-0.48
Apelin (I) & WC (II)	0.35	Follistatin (II) & DBP (II)	-0.34	Follistatin (I) & B-CPX DBPmax	-0.65
Apelin (I) & HC (II)	0.30	Follistatin (II) & 6MWT SBPmax	-0.28	FSTL1 (I) & B-CPX SBPmax	-0.58
Follistatin (I) & HR (I)	0.22	Apelin (I) & 6MWT SO2	-0.40	Apelin (I) & B-CPX SBPmax	0.59
Follistatin (II) & DBP (I)	-0.22	Follistatin (I) & 6MWT total distance walked	-0.25		

Data presented as the Spearman correlation coefficient R value. 6MWT: six minute walk test; B-CPX: cardiac stress test according to Bruce's protocol; BMI: body mass index; BP: blood pressure; DBP: diastolic blood pressure; DBPmax: maximum diastolic blood pressure; FSTL1: follistatin-related protein 1; GDF 8: myostatin; HC: hip circumference; HR heart rate; SBP: systolic blood pressure; SBPmax: maximum systolic blood pressure; SO2: blood oxygen saturation; WC: waist circumference; (I): value before intervention; (II): value after the intervention.
