# Supplementary material for: Cardiac rehabilitation influences serum myokine levels in patients after acute coronary syndrome: the randomised CARDIO-REH study
Source: Sci Rep. 2025 Nov 6;15:38951. doi: 10.1038/s41598-025-22897-0 (PMC12592514; doi:10.1038/s41598-025-22897-0)
Supplement: Supplementary file 6 — Supplementary Material 6 [file 41598_2025_22897_MOESM6_ESM.pdf]

Title: Cardiac rehabilitation influences serum myokine levels in patients after acute coronary syndrome: the randomised CARDIO-REH study
Authors: Damian Skrypnik; Katarzyna Skrypnik; José Casaña Granell; Dawid Woszczyk; Joanna Suliburska
Scientific Reports

Supplementary Table 5. Significant correlations between registered parameters in group K

**Correlations between biochemical parameters**					
GDF 8 & Follistatin	0.39	GDF 8 & Apelin	-0.48	FSTL1 & Apelin	0.32
GDF 8 & FSTL1	-0.25	Follistatin & Apelin	-0.37		
**Correlations between biochemical parameters and anthropometric, body composition parameters, BP and HR**					
GDF 8 & FTC	0.34	Apelin & SBP	-0.30	Follistatin & SBP	0.27
GDF 8 & SBP	0.25	Follistatin & body mass	0.28	FSTL1 & age	0.24
Apelin & body mass	-0.22	Follistatin & BMI	0.21	FSTL1 & %FTC	-0.31
Apelin & HC	-0.31	Follistatin & WC	0.23	FSTL1 & FTC	-0.33

Data presented as the Spearman correlation coefficient R value. %FTC: percentage fat tissue content; BMI: body mass index; FSTL1: follistatin-related protein 1; FTC: mass fat tissue content; GDF 8: myostatin; HC: hip circumference; SBP: systolic blood pressure; WC: waist circumference.
