# Supplementary material for: Cardiac rehabilitation influences serum myokine levels in patients after acute coronary syndrome: the randomised CARDIO-REH study
Source: Sci Rep. 2025 Nov 6;15:38951. doi: 10.1038/s41598-025-22897-0 (PMC12592514; doi:10.1038/s41598-025-22897-0)
Supplement: Supplementary file 7 — Supplementary Material 7 [file 41598_2025_22897_MOESM7_ESM.pdf]

Title: Cardiac rehabilitation influences serum myokine levels in patients after acute coronary syndrome: the randomised CARDIO-REH study

Authors: Damian Skrypnik; Katarzyna Skrypnik; José Casaña Granell; Dawid Woszczyk; Joanna Suliburska

Scientific Reports

Supplementary Table 6A. The regression model ($y = \beta_1 x + \beta_0$) of the relationship between CV risk parameter(x) and **apelin**(y) serum level

**Regression model parameter**	**Group S before the intervention** β_0 = 927.30 R=0.24 R^2=0.06	**Group S after the intervention** β_0 = 11130.19 R=0.44 R^2=0.2	**Group K** β_0 = 1447.26 R=0.58 R^2=0.34
**Resting heart rate (HR)**			
**β_1**	2.69	-113.30	-1.33
**SE**	5.14	93.20	5.23
**p**	0.6038	0.2350	0.8008
**Resting systolic blood pressure (SBP)**			
**β_1**	-0.43	28.37	**-8.61**
**SE**	2.39	39.89	**2.65**
**p**	0.8568	0.4833	**0.0032**
**Resting diastolic blood pressure (DBP)**			
**β_1**	-2.14	-67.79	7.31
**SE**	3.42	72.80	4.43
**p**	0.5339	0.3604	0.1107
**Body mass**			
**β_1**	11.88	-61.64	-4.37
**SE**	19.32	410.86	20.40
**p**	0.5420	0.8819	0.8321
**Body mass index (BMI)**			
**β_1**	-11.16	125.65	0.70
**SE**	23.18	435.54	25.30
**p**	0.6326	0.7753	0.9781

**Percentage fat tissue content (%FTC)**			
**β_1**	-6.46	-69.77	3.48
**SE**	17.54	373.49	26.81
**p**	0.7145	0.8533	0.8976
**Muscle mass (MM)**			
**β_1**	-26.74	165.48	2.80
**SE**	40.86	851.27	50.94
**p**	0.5163	0.8474	0.9566
**Metabolic equivalent of task (MET) in exercise test**			
**β_1**	13.01		
**SE**	20.31		
**p**	0.5251		

CV: cardiovascular; R: correlation coefficient; R^2: R squared; SE: standard error.
