# Supplementary material for: Cardiac rehabilitation influences serum myokine levels in patients after acute coronary syndrome: the randomised CARDIO-REH study
Source: Sci Rep. 2025 Nov 6;15:38951. doi: 10.1038/s41598-025-22897-0 (PMC12592514; doi:10.1038/s41598-025-22897-0)
Supplement: Supplementary file 8 — Supplementary Material 8 [file 41598_2025_22897_MOESM8_ESM.pdf]

**Title:** Cardiac rehabilitation influences serum myokine levels in patients after acute coronary syndrome: the randomised CARDIO-REH study
**Authors**: Damian Skrypnik; Katarzyna Skrypnik; José Casaña Granell; Dawid Woszczyk; Joanna Suliburska
*Scientific Reports*

**Supplementary Table 6B**. The regression model ($y = \beta_1 x + \beta_0$) of the relationship between CV risk parameter(x) and **myostatin**(y) serum level

| Regression model parameter | Group S before the intervention $\beta_0$=-12576.0 R=0.34 $R^2$=0.12 | Group S after the intervention $\beta_0$=8173.86 R=0.25 $R^2$=0.06 | Group K $\beta_0$=-1107.18 R=0.66 $R^2$=0.44 |
|---|---|---|---|
| **Resting heart rate (HR)** | | | |
| $\beta_1$ | 105.9 | -98.45 | 13.71 |
| **SE** | 68.66 | 127.69 | 8.19 |
| **p** | 0.1296 | 0.4462 | 0.1054 |
| **Resting systolic blood pressure (SBP)** | | | |
| $\beta_1$ | 5.8 | 15.46 | 1.35 |
| **SE** | 32.67 | 60.56 | 4.28 |
| **p** | 0.8605 | 0.8002 | 0.7543 |
| **Resting diastolic blood pressure (DBP)** | | | |
| $\beta_1$ | -79.5 | -24.46 | 8.62 |
| **SE** | 46.11 | 111.93 | 7.34 |
| **p** | 0.0911 | 0.8284 | 0.2503 |
| **Body mass** | | | |
| $\beta_1$ | -197.1 | 144.44 | 6.71 |
| **SE** | 264.35 | 548.08 | 31.22 |
| **p** | 0.4595 | 0.7938 | 0.8315 |
| **Body mass index (BMI)** | | | |
| $\beta_1$ | -213.2 | -102.38 | 79.36 |
| **SE** | 352.63 | 598.87 | 40.54 |
| **p** | 0.5483 | 0.8653 | 0.0603 |

| | | | |
|---|---|---|---|
| **Percentage fat tissue content (%FTC)** | | | |
| **$\beta_1$** | 370.8 | -20.77 | -43.19 |
| **SE** | 272.41 | 541.01 | 41.21 |
| **p** | 0.1798 | 0.9696 | 0.3036 |
| **Muscle mass (MM)** | | | |
| **$\beta_1$** | 716.3 | -255.93 | -67.76 |
| **SE** | 587.79 | 1197.09 | 77.60 |
| **p** | 0.2290 | 0.8320 | 0.3900 |
| **Metabolic equivalent of task (MET) in exercise test** | | | |
| **$\beta_1$** | 21.0 | | |
| **SE** | 248.55 | | |
| **p** | 0.9331 | | |

CV: cardiovascular; R: correlation coefficient; $R^2$: R squared; SE: standard error.
