# Supplementary material for: Cardiac rehabilitation influences serum myokine levels in patients after acute coronary syndrome: the randomised CARDIO-REH study
Source: Sci Rep. 2025 Nov 6;15:38951. doi: 10.1038/s41598-025-22897-0 (PMC12592514; doi:10.1038/s41598-025-22897-0)
Supplement: Supplementary file 9 — Supplementary Material 9 [file 41598_2025_22897_MOESM9_ESM.pdf]

Title: Cardiac rehabilitation influences serum myokine levels in patients after acute coronary syndrome: the randomised CARDIO-REH study
Authors: Damian Skrypnik; Katarzyna Skrypnik; José Casaña Granell; Dawid Woszczyk; Joanna Suliburska
Scientific Reports

Supplementary Table 6C. The regression model ($y = \beta_1 x + \beta_0$) of the relationship between CV risk parameter(x) and **follistatin**(y) serum level

**Regression model parameter**	**Group S before the intervention** β_0=-24.27 R=0.45 R^2=0.2	**Group S after the intervention** β_0=9.11 R=0.55 R^2=0.3	**Group K** β_0=11.54 R=0.37 R^2=0.14
**Resting heart rate (HR)**			
**β_1**	0.05	0.02	0.04
**SE**	0.12	0.10	0.09
**p**	0.7045	0.8855	0.6107
**Resting systolic blood pressure (SBP)**			
**β_1**	0.08	-0.01	0.03
**SE**	0.06	0.05	0.05
**p**	0.1999	0.8436	0.5871
**Resting diastolic blood pressure (DBP)**			
**β_1**	-0.12	**-0.19**	-0.01
**SE**	0.08	**0.09**	0.08
**p**	0.1586	**0.0430**	0.9422
**Body mass**			
**β_1**	-0.32	-0.15	0.36
**SE**	0.47	0.44	0.35
**p**	0.4994	0.7418	0.3055
**Body mass index (BMI)**			
**β_1**	0.31	0.43	0.11
**SE**	0.57	0.49	0.45
**p**	0.5882	0.3785	0.8032

**Percentage fat tissue content (%FTC)**			
**β_1**	0.34	-0.01	-0.43
**SE**	0.43	0.44	0.45
**p**	0.4360	0.9827	0.3463
**Muscle mass (MM)**			
**β_1**	1.17	0.46	-0.95
**SE**	0.99	0.97	0.86
**p**	0.2420	0.6370	0.2760
**Metabolic equivalent of task (MET) in exercise test**			
**β_1**	-0.33		
**SE**	0.45		
**p**	0.4658		

CV: cardiovascular; R: correlation coefficient; R^2: R squared; SE: standard error.
