# Supplementary material for: Cardiac rehabilitation influences serum myokine levels in patients after acute coronary syndrome: the randomised CARDIO-REH study
Source: Sci Rep. 2025 Nov 6;15:38951. doi: 10.1038/s41598-025-22897-0 (PMC12592514; doi:10.1038/s41598-025-22897-0)
Supplement: Supplementary file 10 — Supplementary Material 10 [file 41598_2025_22897_MOESM10_ESM.pdf]

Title: Cardiac rehabilitation influences serum myokine levels in patients after acute coronary syndrome: the randomised CARDIO-REH study
Authors: Damian Skrypnik; Katarzyna Skrypnik; José Casaña Granell; Dawid Woszczyk; Joanna Suliburska
Scientific Reports

Supplementary Table 6D. The regression model ($y = \beta_1 x + \beta_0$) of the relationship between CV risk parameter (x) and **follistatin-related protein 1** (y) serum level

**Regression model parameter**	**Group S before the intervention** β_0 = -269.59 R= 0.44 R^2= 0.19	**Group S after the intervention** β_0 = 96.57 R= 0.72 R^2= 10.66	**Group K** β_0 = 160.76 R= 0.38 R^2= 0.14
**Resting heart rate (HR)**			
**β_1**	0.54	0.28	-1.16
**SE**	1.33	0.54	0.81
**p**	0.6848	0.6043	0.1615
**Resting systolic blood pressure (SBP)**			
**β_1**	0.17	-0.06	-0.59
**SE**	0.62	0.25	0.43
**p**	0.7901	0.8107	0.1829
**Resting diastolic blood pressure (DBP)**			
**β_1**	-0.91	-0.21	0.42
**SE**	0.89	0.47	0.70
**p**	0.3119	0.6540	0.5523
**Body mass**			
**β_1**	-4.28	**5.14**	0.82
**SE**	4.94	**2.30**	3.26
**p**	0.3903	**0.0326**	0.8024
**Body mass index (BMI)**			
**β_1**	8.03	**7.54**	3.30
**SE**	6.00	**2.52**	4.14
**p**	0.1866	**0.0051**	0.4314

**Percentage fat tissue content (%FTC)**			
**β_1**	3.72	**-7.61**	-2.88
**SE**	4.55	**2.27**	4.32
**p**	0.4173	**0.0020**	0.5099
**Muscle mass (MM)**			
**β_1**	10.66	**-15.11**	-2.94
**SE**	10.53	**5.03**	8.17
**p**	0.3166	**0.0051**	0.7208
**Metabolic equivalent of task (MET) in exercise test**			
**β_1**	-2.50		
**SE**	5.10		
**p**	0.6263		

CV: cardiovascular; R: correlation coefficient; R^2: R squared; SE: standard error.
